# Reduction of Dislocation of the Femur on the Dorsum of the Ilium, after the Plan of Dr. Reid

**Published:** 1851-10

**Authors:** 


*Reduction of Dislocation of the Femur on the Dorsum of the Ilium, after the Plan of Dr. Reid.*

Our readers will observe in the present Number a report of a case in which the new plan of reducing dislocation of the femur, was found to be eminently successful. If we do not greatly err, the publication of the article by Dr. Reid will prove to be an important epoch in practical surgery. An anonymous writer in the Boston Medical and Surgical Journal, (who states that he does not practice surgery,) claims that the manipulations practiced by Dr. Reid are devoid of novelty, having been recommended, many years ago, by the distinguished Nathan Smith. The careful reader of Dr. Reid's article will perceive that he gives credit to Dr. Smith for a plan which he suspects may have been somewhat analogous. This, however, does not in the least detract from the merit, or utility of the investigations by Dr. Reid. It cannot be denied that Dr. Reid's plan is an innovation in the course of practice recommended by the best writers, and pursued by the ablest practical surgeons of the present time. Now the question is, does the plan possess the advantages claimed. If it does, Dr. Reid's name will ever be associated with one of the most important improvements in surgical science which has been effected in our day.

